# Increasing Angiogenesis Factors in Hypoxic Diabetic Wound Conditions by siRNA Delivery: Additive Effect of LbL-Gold Nanocarriers and Desloratadine-Induced Lysosomal Escape

**DOI:** 10.3390/ijms22179216

**Published:** 2021-08-26

**Authors:** Elnaz Shaabani, Maryam Sharifiaghdam, Joris Lammens, Herlinde De Keersmaecker, Chris Vervaet, Thomas De Beer, Elahe Motevaseli, Mohammad Hossein Ghahremani, Parvin Mansouri, Stefaan De Smedt, Koen Raemdonck, Reza Faridi-Majidi, Kevin Braeckmans, Juan C. Fraire

**Affiliations:** 1 Laboratory of General Biochemistry and Physical Pharmacy, Faculty of Pharmacy, Ghent University, Ottergemsesteenweg 460, 9000 Ghent, Belgium; elnaz.shaabanisichani@ugent.be (E.S.); maryam.sharifiaghdam@ugent.be (M.S.); herlinde.dekeersmaecker@ugent.be (H.D.K.); Stefaan.Desmedt@ugent.be (S.D.S.); koen.raemdonck@UGent.be (K.R.); juan.fraire@ugent.be (J.C.F.); 2 Department of Medical Nanotechnology, School of Advanced Technologies in Medicine, Tehran University of Medical Sciences, Tehran, Iran; 3 Laboratory of Pharmaceutical Technology, Department of Pharmaceutics, Ghent University, Ottergemsesteenweg 460, 9000 Ghent, Belgium; joris.lammens@ugent.be (J.L.); Chris.Vervaet@ugent.be (C.V.); 4 Center for Advanced Light Microscopy, Ghent University, 9000 Ghent, Belgium; 5 Laboratory of Pharmaceutical Process Analytical Technology (LPPAT), Department of Pharmaceutical Analysis, Ghent University, Ottergemsesteenweg 460, 9000 Ghent, Belgium; Thomas.DeBeer@Ugent.be; 6 Department of Molecular Medicine, School of Advanced Technologies in Medicine, Tehran University of Medical Sciences, Tehran, Iran; e_motevaseli@tums.ac.ir; 7 Department of Toxicology and Pharmacology, Faculty of Pharmacy, Tehran University of Medical Sciences, Tehran, Iran; mhghahremani@tums.ac.ir; 8 Skin and Stem Cell Research Center, Tehran University of Medical Sciences, Tehran, Iran; mansorip@sina.tums.ac.ir

**Keywords:** diabetic wound healing, hypoxia, angiogenesis, cationic amphiphilic drugs, gold nanoparticles, gene delivery, layer-by-layer

## Abstract

Impaired wound healing in people with diabetes has multifactorial causes, with insufficient neovascularization being one of the most important. Hypoxia-inducible factor-1 (HIF-1) plays a central role in the hypoxia-induced response by activating angiogenesis factors. As its activity is under precise regulatory control of prolyl-hydroxylase domain 2 (PHD-2), downregulation of PHD-2 by small interfering RNA (siRNA) could stabilize HIF-1α and, therefore, upregulate the expression of pro-angiogenic factors as well. Intracellular delivery of siRNA can be achieved with nanocarriers that must fulfill several requirements, including high stability, low toxicity, and high transfection efficiency. Here, we designed and compared the performance of layer-by-layer self-assembled siRNA-loaded gold nanoparticles with two different outer layers—Chitosan (AuNP@CS) and Poly L-arginine (AuNP@PLA). Although both formulations have exactly the same core, we find that a PLA outer layer improves the endosomal escape of siRNA, and therefore, transfection efficiency, after endocytic uptake in NIH-3T3 cells. Furthermore, we found that endosomal escape of AuNP@PLA could be improved further when cells were additionally treated with desloratadine, thus outperforming commercial reagents such as Lipofectamine^®^ and jetPRIME^®^. AuNP@PLA in combination with desloratadine was proven to induce PHD-2 silencing in fibroblasts, allowing upregulation of pro-angiogenic pathways. This finding in an in vitro context constitutes a first step towards improving diabetic wound healing with siRNA therapy.

## 1. Introduction

**Scheme 1:**
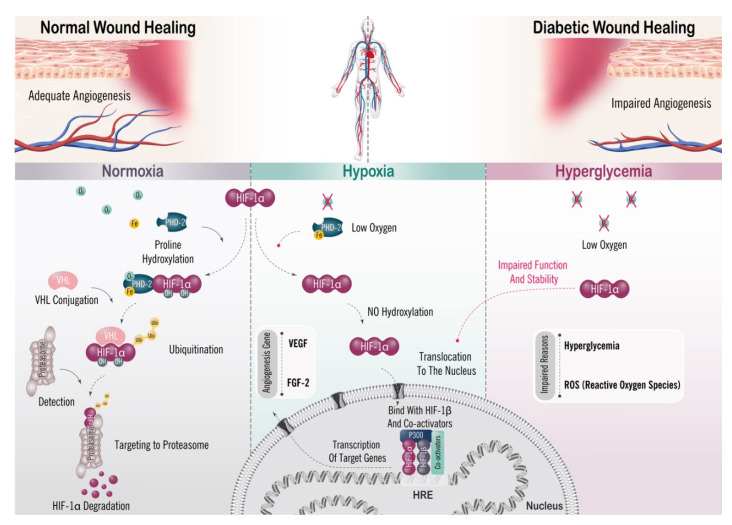
Schematic representation of how hypoxia-inducible factor 1 (HIF-1) activates gene transcription in response to normoxic vs. hypoxic conditions. Under normoxic conditions, HIF-1α is subjected to hydroxylation by prolyl hydroxylase domain protein 2 (PHD-2). This hydroxylation is required for binding of the von Hippel–Lindau protein (VHL), the recognition subunit of a ubiquitin protein ligase that targets HIF-1α for ubiquitination and proteasomal degradation. Under hypoxic conditions, hydroxylation is inhibited and HIF-1α is stabilized, which dimerizes with HIF-1β and binds to target genes at the consensus sequence HRE. The target gene DNA sequence is then transcribed into messenger RNA (mRNA). Under hyperglycemic conditions in the diabetic wound, the function and stability of HIF-1α are impaired by high levels of glucose and reactive oxygen species (ROS), so that fewer new vessels are formed and wound healing becomes impaired.

Diabetes mellitus (DM) is a metabolic chronic disease characterized by hyperglycemia (high levels of glucose in the blood), which results from defective insulin secretion, defective insulin action, or both [1,2,3]. An insulin deficit, in the long term, can cause damage to many organs and tissues, leading to life-threatening health complications such as neuropathy [4,5,6], cardiovascular diseases [7,8,9], nephropathy [10,11,12], ocular diseases [13,14] and ulcerations [15,16,17]. Among those, the development of chronic nonhealing foot ulcerations (diabetic foot ulcer (DFU)) [18], which increases the risk of amputation [19] and is considered to be a major source of mortality, is the leading cause of hospitalization of diabetes patients [20,21]. Clinical and experimental evidence indicates that impaired wound healing in diabetic patients has multifactorial causes due to which the wound healing process can become halted in different phases [22,23,24]. Recent studies have shown that one of the main pathological mechanisms of impaired diabetic wound healing is insufficient or delayed neovascularization [25,26,27]. At the molecular level, the angiogenic process is regulated by hypoxia-inducible factor-1 (HIF-1) [28,29], which mediates the cellular response to hypoxia, and promotes pro-angiogenic gene transcription, thus stimulating neovascularization [30,31]. HIF-1, a member of the heterodimeric transcription factor family, consists of a highly regulated α-subunit (HIF-1α) and constitutively expressed β-subunit (HIF-1β) [29,32]. HIF-1α activity is regulated by the oxoglutarate-dependent prolyl hydroxylase domain-2 (PHD-2) protein. In normoxia, HIF-1α is hydroxylated on proline residues by PHD-2. The hydroxylated form of HIF-1α is required for binding of the von Hippel–Lindau protein (VHL) that is part of an E3 ubiquitin ligase complex, which targets HIF-1α for ubiquitination and proteasomal degradation [33,34,35]. Under hypoxic conditions, the degradation pathway of HIF-1α is suppressed, allowing dimerization with HIF-1β in the nucleus, and binds to the hypoxia response element (HRE), which promotes transcription of a cascade of genes that enhance oxygen delivery such as multiple angiogenic growth factors [36], cell metabolism [37], proliferation [38], and the recruitment of endothelial progenitor cells [39] (Scheme 1). Together, this indicates that HIF-1α plays a major role in the angiogenesis process, requiring high expression levels in normal wounds under hypoxic conditions for wound healing [40]. Despite the diabetic wound environment being hypoxic, many studies have shown that the function and stability of HIF-1α are impaired by hyperglycemia and the presence of reactive oxygen species (ROS) [41,42,43], leading to reduced formation of new blood vessels [43,44]. Therefore, the stabilization of HIF-1α in diabetic wounds could stimulate the expression of hypoxia-inducible genes, in turn, stimulating angiogenesis and accelerating wound healing. This can be accomplished by downregulation of PHD-2, which should result in stabilization, and upregulation of HIF-1α [45,46], which has been proven to improve wound healing in diabetic conditions [47,48,49,50,51].

Activation of the intracellular RNA interference (RNAi) pathway via small interfering RNA (siRNA) is a powerful therapeutic technology to induce post-transcriptional sequence-specific gene silencing [52,53]. Unmodified siRNA faces fast enzymatic digestion, limited cellular uptake, and inefficient release from endosomes if not incorporated into a suitable delivery system [54]. One of the most widely used delivery vectors is cationic polymers, which can easily form polymer–nucleic acid complexes (polyplexes) by electrostatic interactions with negatively charged oligonucleotides [55]. Two polymers are of particular interest due to their biodegradability and biocompatibility, i.e., chitosan (a linear cationic polysaccharide) [56] and poly L-arginine (a linear homopolymer of the basic amino acid L-arginine) [57]. However, it remains challenging to design polymeric siRNA nanocarriers that have good stability in biological tissues while, at the same time, achieving efficient transfection of target cells [58].

**Scheme 2:**
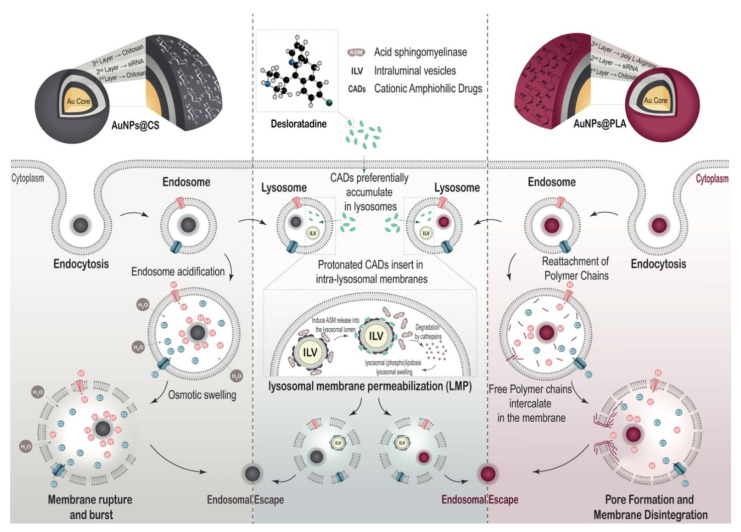
**Schematic representation of AuNP@CS and AuNP@PLA for siRNA delivery.** The NPs are taken up by the cells via endocytosis and are present inside endosomes. Endosomal escape happens through the proton sponge effect in the case of CS, or through membrane pore formation in the case of PLA [88]. NPs routed toward the lysosomal compartment with CAD-induced transient lysosomal membrane permeabilization (LMP) can diffuse from the lysosomal lumen into the cytosol.

After endocytosis in the target cells, it is essential that the carrier can induce efficient endosomal escape. This last step, however, remains one of the major bottlenecks for siRNA delivery systems to date, resulting in the majority of endocytosed nanocarriers being routed toward endolysosomes for degradation [59]. Recently, it was demonstrated that endosomal escape of siRNA-loaded nanocarriers can be enhanced by treating cells with cationic amphiphilic drugs (CADs) [60,61]. Due to the physicochemical properties of CADs, they are able to accumulate inside the acidified lysosomal compartment in their protonated form. The cationic lysosomal membrane-associated enzyme acid sphingomyelinase (ASM) is electrostatically bound to the anionic bis(monoacylglycero)phosphate (BMP) lipids of the intraluminal vesicles. Protonated CADs induce ASM detachment and inhibition [62,63], which results in lysosomal membrane permeabilization (LMP) and lysosomal swelling, thus helping to release the siRNA molecules from the endolysosomes into the cytosol [60,61] (Scheme 2).

The main goal of this study is to find an efficient and stable siRNA nanoformulation for silencing PHD-2 in fibroblast as target cells, which should stimulate angiogenesis in diabetic wounds. In a recent study, we explored siRNA nanocarriers consisting of chitosan-coated gold nanoparticles (AuNPs), onto which siRNA was electrostatically complexed and protected by another final layer of chitosan [64]. These layer-by-layer (LbL) nanocarriers demonstrated long-term colloidal stability and stable incorporation of siRNA. Building further on this idea, we evaluated these LbL nanocarriers (AuNP@CS) for PHD-2 downregulation and, in particular, explored the effect of exchanging the final chitosan layer with poly L-arginine (AuNP@PLA). As shown in Scheme 2, both polymers have distinct endosomal escape mechanisms. The buffering capacity of chitosan leads to osmotic swelling and disruption of the endosome [65], while poly L-arginine (PLA) binds to the endosome’s lipid bilayer, leading to endosomal escape by pore formation [66,67,68,69]. After synthesis and detailed physicochemical characterization of AuNP@CS and AuNP@PLA, cellular cytotoxicity, internalization and endosomal escape were studied in NIH-3T3 fibroblast cells and compared with commercial transfection agents (Lipofectamine^®^ RNAiMAX and jetPRIME^®^). Next, we explored the combination of those nanocarriers with the CAD molecule desloratadine (DES) in terms of transfection efficiency, downstream gene expression, proliferation and cell migration. Finally, downregulation of PHD-2 was investigated in NIH-3T3 fibroblasts, as well as upregulation of VEGF and FGF (angiogenesis and neovascularization factors), which can accelerate diabetic wound healing.

## 2. Results

### 2.1. Synthesis and Physicochemical Evaluation of Nanoformulations

**Figure 1:**
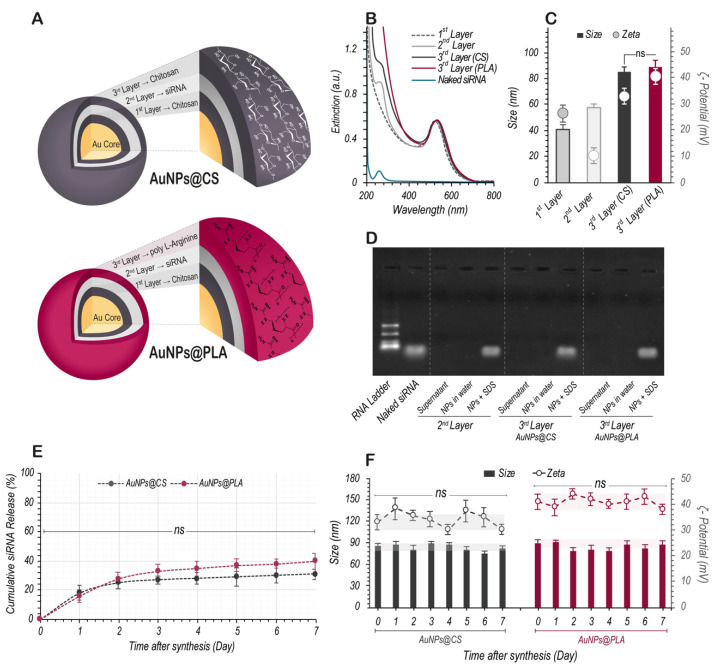
**Characterization of layer-by-layer (LbL) synthesis of siRNA-AuNP nanoformulations with CS or PLA as outer layer.** (**A**) Schematic representation of the LbL nanoformulations with either CS (AuNPs@CS) or PLA (AuNPs@PLA) as the 3rd and final layer. (**B**) UV–vis spectra upon sequential adsorption of the different layers in the LbL synthesis. (**C**) Particle size and zeta potential measured by DLS after sequential adsorption of the different layers in the LbL synthesis. (**D**) Agarose gel retardation assay of nanoformulations upon addition of each layer. After centrifugation, the supernatant was collected and the pellet of nanoformulations was redispersed in water or mixed with 20 mg/mL sodium dodecyl sulfate (SDS) for dissociation of siRNA from the nanoparticles. (**E**) Percentage of siRNA released from AuNPs@CS and AuNPs@PLA in pH 7.4 HEPES buffer at 37 °C as a function of time. (**F**) Size and zeta potential analysis in ddi water as a function of time for AuNPs@CS and AuNPs@PLA. Data are represented as mean ± the standard deviation of the mean for a minimum of three independent experiments (ns = not significant).

As reported previously [64], to obtain a stable siRNA carrier, a layer-by-layer (LbL) self-assembly technique was used through sequential electrostatic adsorption of oppositely charged polymers onto a solid AuNP core. Spherical AuNPs were synthesized using the positively charged chitosan (CS), which acts as both a reducing and capping agent and which forms a first layer (Figure 1A). Next, negatively charged siRNA is added as a second layer in a 1:10 siRNA:Au ratio. Finally, a third positively charged layer of chitosan (AuNP@CS) or poly L-arginine (AuNP@PLA) was applied to protect the siRNA and facilitate endocytic uptake of the carriers.

This process of layer-by-layer deposition was monitored by measuring the UV–vis spectra (Figure 1B) as well as the hydrodynamic diameter and zeta potential with dynamic light scattering (DLS; Figure 1C). The initial chitosan-coated AuNPs showed a localized surface plasmon resonance (LSPR) peak at 524 nm, confirming the formation of AuNPs. Their size was 40 ± 5 nm and zeta potential 26 ± 3 mV. siRNA absorbs UV light at a wavelength of 260 nm due to the resonance structure of the purine and pyrimidine bases [70]. This absorption band was indeed visible for naked siRNA and also appeared in the nanoformulation’s spectrum after the second layer was applied. Upon adding siRNA to the formulation, the size increased to 57 ± 3 nm, while the zeta potential reduced to 10 ± 3 mV. Upon addition of chitosan as the third and last layer, the size and zeta potential increased to 86 ± 4 nm and 33 ± 3 mV, respectively. Poly L-arginine has substantial absorption between 250 and 400 nm [71] (Figure S1A), resulting in a further increase in the UV–vis spectrum in that range after applying PLA as a third layer. The size increased to 88 ± 5 nm, while the zeta potential became strongly positive at 41 ± 3 mV.

To further confirm the deposition of the 3rd layer on the surface of AuNPs, we characterized the nanoformulations by Fourier-transform infrared (FTIR) spectroscopy, as shown in Figure S1C. The characteristic bands of chitosan (1558 cm^−1^ = N–H stretching vibration, 1398 cm^−1^ and 2879 cm^−1^ = C–H vibration and 1027 cm^−1^ = C–O stretching vibration) [72] or poly L-arginine (1660 cm^−1^ = amide I (C=O carbonyl stretch and guanidine C=N stretch), 1544 cm^−1^ = amide II (C=N stretch and N–H bending) 1037 cm^−1^ = C–N stretching vibration and 3290 cm^−1^ = N–H stretching vibration) [73,74] could indeed be observed in the AuNP@CS and AuNP@PLA infrared spectrums, respectively.

Successful siRNA complexation was additionally evaluated by gel electrophoresis (Figure 1D), where the electrophoretic runs revealed no band of free siRNA in the supernatant, indicating that all siRNA was successfully complexed to the nanoparticles and confirming that the siRNA did not dissociate after applying the third layer. When the nanoformulations were incubated with sodium dodecyl sulfate (SDS-30 mg/mL), whose negative charge can displace the incorporated siRNA, a band of siRNA could again be observed. Note that the signal intensity of the siRNA bands of AuNP@CS and AuNP@PLA is almost identical, indicating that equal amounts of siRNA were loaded in both formulations.

Next, we monitored siRNA release from the nanoformulations in pH 7.4 HEPES buffer over time. Both formulations had a virtually identical release profile, characterized by a fairly quick release of 30% siRNA during the first two days and a slow release of another 10% over the next 5 days (Figure 1E). Finally, we evaluated the size and zeta potential of the nanoformulations over time in water (Figure 1F). Both AuNP@CS and AuNP@PLA remained stable for at least 7 days without significant changes in zeta potential or size. Additionally, the UV–vis spectrum remained unchanged after 7 days (Figure S1B).

### 2.2. Cellular Toxicity and Internalization of Nanoformulations

Fibroblast cells are critical in the wound healing process, from the late inflammatory phase until the full final epithelization by secreting growth factors and cytokines, creating new extracellular matrix (ECM) and collagen structures, acting as a support and signal for angiogenesis and re-epithelialization [75,76]. Therefore, NIH-3T3 murine fibroblast cells were chosen as an in vitro model for this study. To simulate the hyperglycemic conditions in diabetic wounds, cells were cultured in medium supplemented with approximately 4500 mg/L D-glucose, which approximates the diabetic levels of glucose in vivo [77,78].

**Figure 2:**
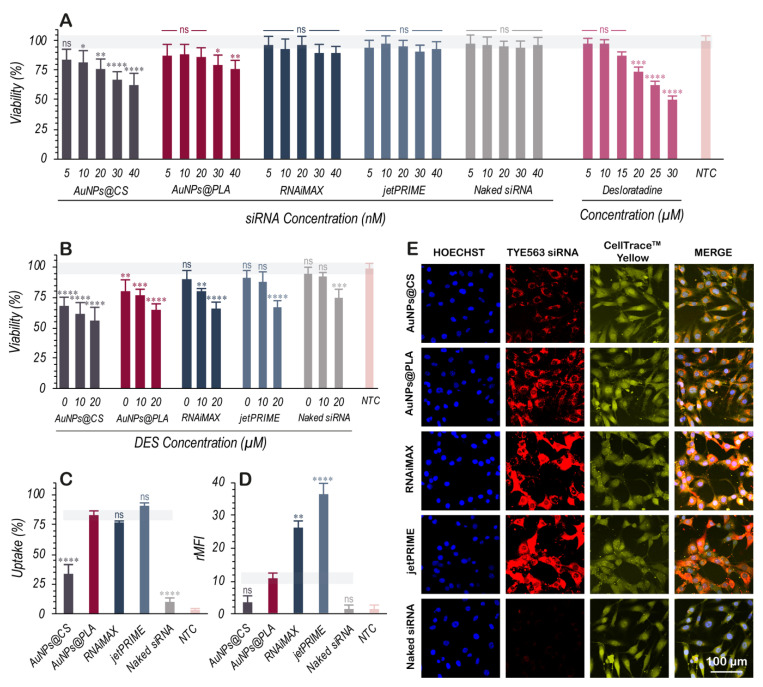
**Cytotoxicity and evaluation of nanoformulation internalization in NIH-3T3 cells.** (**A**) Cell viability of NIH-3T3 cells was measured by CellTiter GLO^®^ after 4 h incubation with AuNPs@CS, AuNPs@PLA, RNAiMAX, jetPRIME^®^, and Naked siRNA at different concentrations (5, 10, 20, 30, and 40 nM of siRNA). The cytotoxic effect of 20 h incubation with DES at different concentrations (5, 10, 15, 20, 25, and 30 µM) was tested as well. (**B**) Cell viability of NIH-3T3 cells after 4 h incubation with the nanoformulations and transfection reagents (30 nM effective concentration of siRNA), washing and 20 h incubation with DES (0, 10, and 20 µM). (**C**) Uptake percentage (=% positive cells) and (**D**) relative mean fluorescence intensity per cell of TYE563 siRNA-labeled nanoformulations and transfection reagents (30 nM) after 4 h treatment on NIH-3T3 cells as determined by flow cytometry. (**E**) Confocal images of NIH-3T3 cells after 4 h treatment with different nanoformulations and transfection reagents at 30 nM effective concentration of siRNA. Nuclei were stained with Hoechst 33342 (blue), the cytoplasm with CellTrace™ Yellow (yellow), while TYE563 siRNA is shown in red. The scale bar corresponds to 100 μm. Data are represented as the mean ± the standard deviation for a minimum of three independent experiments. Statistical significance is indicated when appropriate, in (**A**,**B**) when referring to not treated control (NTC) and in (**C**,**D**) when compared to AuNPs@PLA. (ns = not significant *p* > 0.05, * *p* ≤ 0.05, ** *p* ≤ 0.01, *** *p* ≤ 0.001, **** *p* ≤ 0.0001).

First, the dose-dependent toxicity of our nanoformulations was studied on NIH-3T3 by measuring the cell’s metabolic activity (via CellTiter-Glo^®^ luminescent assay). Cells were incubated with nanoparticles (NPs) for 4 h, followed by 20 h incubation in fresh cell culture medium. The relation between particle and siRNA concentration is shown in Table S1. Lipofectamine^®^ RNAiMAX and jetPRIME^®^, two widely used commercial siRNA transfection reagents, were included in all experiments for benchmarking. We also checked the toxicity induced by increasing concentrations of desloratadine (DES), which we will evaluate as CAD molecule for enhancing endosomal escape further on. As can be seen in Figure 2A, toxicity increases with increasing NP concentration, with AuNP@PLA being somewhat less toxic than AuNP@CS. For the same siRNA concentrations, Lipofectamine^®^ RNAiMAX, jetPRIME^®^ and naked siRNA did not induce any significant toxic effects. Considering 30% loss of metabolic activity as a commonly chosen acceptable level of cytotoxicity, 30 nM was selected as the highest allowed concentration for our nanoformulations. Treating NIH-3T3 cells with DES likewise caused a concentration-dependent toxicity, from which 20 µM DES was selected as the maximal concentration to be used.

We also evaluated the effect of combining NP treatment with DES on cell viability. Similar to a previous study where DES was used to enhance endosomal escape of siRNA nanocarriers [61], cells were incubated with NPs or commercial transfection reagents for 4 h, after which cells were washed and further incubated in the presence of DES-supplemented culture medium for 20 h. Figure 2B (30 nM of siRNA) and Figure S2A (10 nM of siRNA) indicate that the addition of DES reduced viability in a concentration-dependent manner compared to NPs or transfection reagents alone, but overall, it was well tolerated in the applied concentration range (10 and 20 µM) for all nanoformulations.

Next, we performed flow cytometry measurements (Figure 2C,D and Figure S2B,C) and confocal microscopy imaging (Figure 2E and Figure S2D) to measure and visualize the uptake of NPs and transfection reagents at 10 nM and 30 nM siRNA concentrations. For these experiments, fluorescent TYE 563-labeled siRNA (TYE563 siRNA) was used. As shown in Figure 2C,D for 30 nM siRNA, the percentage of cells positive for AuNP@PLA was substantially higher (83 ± 4%) as compared to AuNP@CS (34 ± 8%). This indicates that the PLA outer layer has a greater capacity to facilitate uptake than CS [79]. With RNAiMAX and jetPRIME^®^, similar levels of positive cells were obtained, but with much higher relative mean fluorescent intensity (rMFI), showing that they can induce more uptake per cell. These findings can be visually appreciated from the confocal images as well where Hoechst was used to stain the nuclei (blue) and CellTraceTM Yellow to stain the cytoplasm (Figure 2E). Identical experiments were performed for a lower siRNA concentration of 10 nM, essentially showing the same relative trends (Figure S2).

### 2.3. Lysosomal Swelling by DES and Its Effect on Endosomal Escape in NIH-3T3 Cells

**Figure 3:**
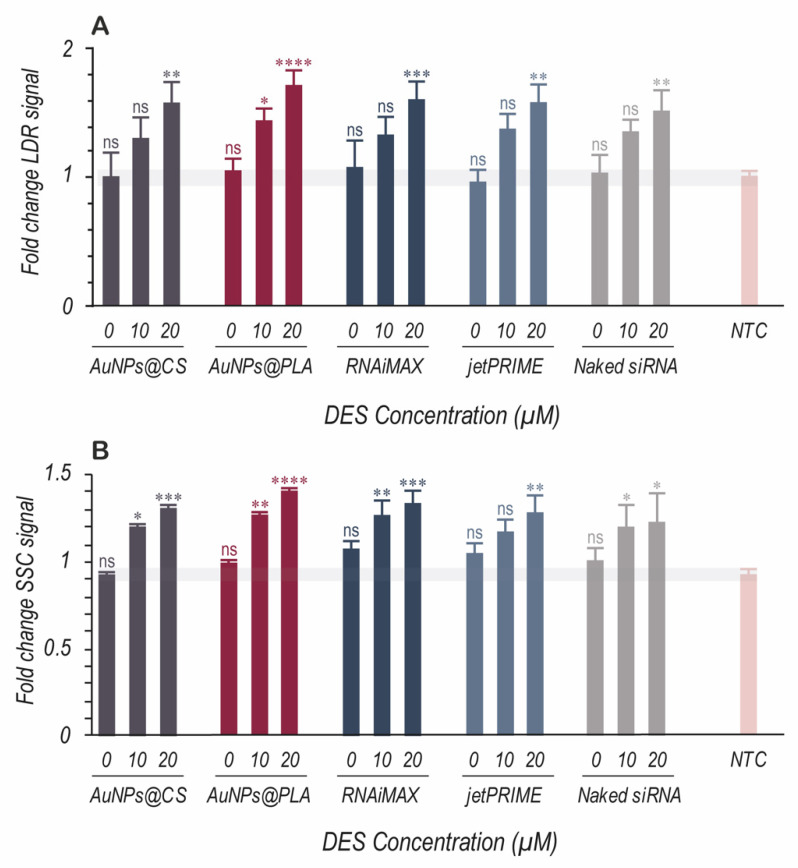
**The effect of DES on lysosomal swelling and cellular granularity.** (**A**) Fold change in LysoTracker^®^ Deep Red (LDR) signal and (**B**) Fold change in side scatter (SSC) signal were measured via flow cytometry for NIH-3T3 cells treated 4 h with nanocarriers (30 nM effective concentration of siRNA) followed by 20 h treatment with 3 different concentrations of DES (0, 10, and 20 μM). Data are represented as the mean ± the standard deviation of three independent experiments. Statistical significance, with respect to the not treated control (NTC), is indicated when appropriate (ns = not significant *p* > 0.05, * *p* ≤ 0.05, ** *p* ≤ 0.01, *** *p* ≤ 0.001, **** *p* ≤ 0.0001).

Previous studies have shown that cells exposed to CADs present lysosomal volume expansion and an increase in cellular granularity. This was found for several human cell lines, including non-small cell lung epithelial carcinoma cells stably expressing eGFP (H1299-eGFP), ovarian cancer cells stably expressing pGL3 firefly luciferase (SKOV-3-LUC+), and HeLa cells stably transfected with a GFP-tagged nuclear-localization signal (HeLa NLS-GFP) [60,61]. To see if the same effects apply to NIH-3T3 fibroblasts, we made use of the LysoTracker^®^ Deep Red (LDR) probe. LDR is a deep red fluorescent dye that can accumulate in acidic organelles such as lysosomes. If CAD-induced lysosomal swelling occurs, more LDR dye will accumulate in the lysosomes and increase the red fluorescence intensity when measured by flow cytometry. In addition, lysosomal swelling will increase cellular granularity, which can be detected by an augmented side scatter (SSC) signal in flow cytometry. As can be seen in Figure 3 (30 nM of siRNA) and Figure S3 (10 nM of siRNA), for all nanoformulations and transfection reagents, DES evoked a similar concentration-dependent LDR and SCC signal increase compared to non-treated control (NTC) cells. In all cases, the highest DES concentration (20 μM) resulted in a significantly higher LDR and SSC signal. The change in SSC signal can also be appreciated from the representative flow cytometry contour plots in Figure S4.

**Figure 4:**
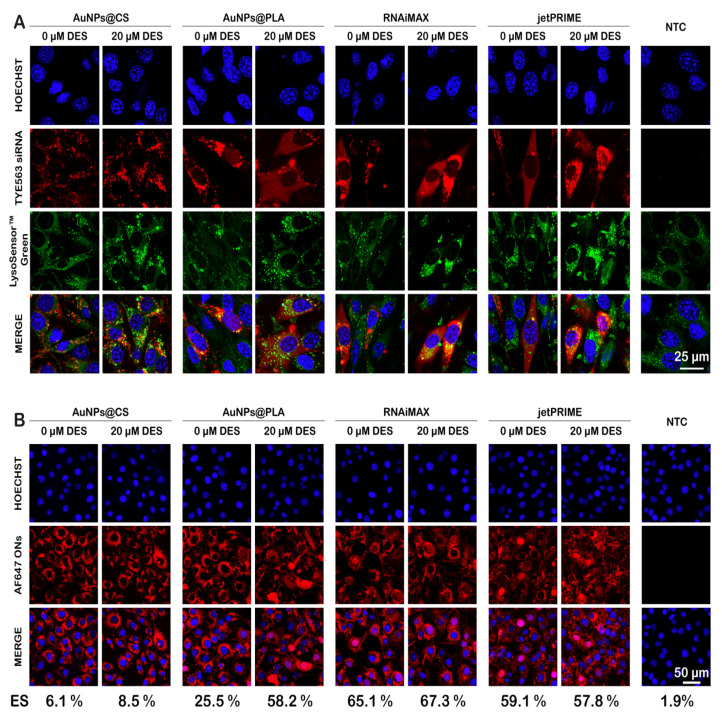
**Uptake and endosomal escape of nanoformulations alone or in combination with DES.** (**A**) Representative confocal images of NIH-3T3 cells incubated 4 h with TYE563 siRNA-loaded nanoformulations (red), followed by 20 h incubation with cell medium with or without 20 μM DES. Hoechst-stained nuclei can be seen in blue, while late endosomes and lysosomes are stained with LysoSensorTM green. The scale bar corresponds to 25 μm. (**B**) Representative confocal images of NIH-3T3 cells, incubated 4 h with AF647 ON-loaded carriers, followed by 20 h incubation with cell medium with or without 20 μM DES. Hoechst-stained nuclei can be seen in blue, while cells in which endosomal escape happened have red fluorescent nuclei due to the release of AF647 ONs, which accumulate in the nucleus. The values below the images correspond to the percentage of cells with red nuclei (endosomal escape (ES)), as determined from at least 500 cells for each case. The scale bar corresponds to 50 μm.

This lysosomal swelling is believed to improve cytosolic delivery of siRNA through the induction of lysosomal membrane permeabilization (LMP). To investigate if this is the case, we loaded TYE563 siRNA into the carriers and stained the lysosomal compartments with LysoSensorTM green. Confocal images showed that AuNP@CS resulted in a dotted pattern in the red channel, which colocalized strongly with endolysosomes, indicating that most of those particles remain trapped inside the endolysosomes (Figure 4A). This was different for AuNP@PLA in combination with DES, Lipofectamine^®^ RNAiMAX, and jetPRIME^®^, for which a more diffuse signal in the cytosol was observed. Note that for DES-treated groups, a clear enlargement in LysoSensorTM green-labeled vesicles could be observed, thus confirming the flow cytometry results.

To further confirm endosomal escape, we prepared similar nanoformulations, in which siRNA was exchanged for AF674-labeled oligonucleotides (ONs). Upon endo/lysosomal escape, ONs will migrate to the cell nucleus by active transport, leading to a red fluorescent nucleus. By counting the total number of nuclei in the confocal images (detected by Hoechst staining) and red fluorescent nuclei (due to endosomal escape of the ONs), the percentage of cells in which endo/lysosomal escape events have occurred can be determined [64,80]. Representative microscopy images of NIH-3T3 cells after 24 h treatment with NPs loaded with 30 nM ONs can be seen in Figure 4B. The percentage of cells showing endo/lysosomal escape, which is mentioned below the images for each condition, was again found to be considerably lower for AuNP@CS in comparison with the other carriers. Furthermore, upon DES treatment, only AuNP@PLA showed a clear increase in the number of red fluorescent nuclei, while there was no obvious difference for any of the other carriers. Thus, we conclude that DES treatment of cells results in a selective increase in endosomal escape for AuNP@PLA alone.

### 2.4. Forced siRNA Dissociation from the Nanoformulations

To produce a biological effect, it is essential that siRNA is released from the nanocarriers into the cytoplasm. To investigate the relative binding strength of siRNA to the various types of nanoformulations, a forced release assay was performed by adding increasing concentrations of SDS, which induces the release of siRNA. siRNA dissociation from the carriers was measured by gel electrophoresis and fluorescence fluctuation spectroscopy (FFS).

Both techniques demonstrated a gradual release of siRNA with increasing amounts of SDS from all carriers, reaching full dissociation at 30 mg/mL (Figure S5). Release of siRNA from jetPRIME^®^ and AuNP@CS started at a relatively higher concentration of SDS as compared to Lipofectamine^®^ RNAiMAX and AuNP@PLA. A lack of decomplexation may at least in part explain why so little endosomal escape could be observed from AuNP@CS compared to the other formulations.

### 2.5. Downregulation of PHD-2 and Its Effect on HIF-1α Expression for Promoting Upregulation of Angiogenesis Factors

**Figure 5:**
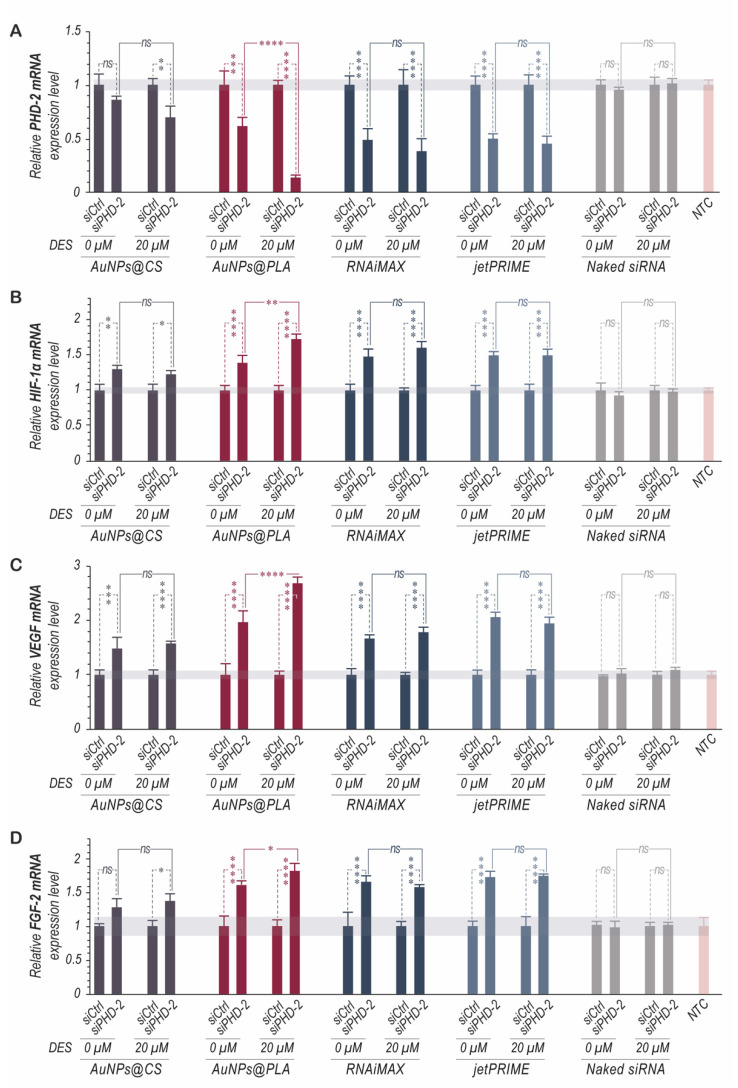
**Knockdown of PHD-2 and its effect on the expression of angiogenic growth factors in NIH-3T3 cells.** NIH-3T3 cells were incubated for 4 h with nanoformulation loaded with 30 nM non-specific siRNA (siCtrl) or siPHD-2, followed by 20 h incubation with cell culture medium without or with 20 µm DES. After another 24 h incubation in fresh cell medium, mRNA was isolated and subjected to real-time reverse transcriptase PCR analysis to determine expression of (**A**) prolyl hydroxylase domain-2 (PHD-2), (**B**) hypoxia inducible factor-1α (HIF-1α), (**C**) vascular endothelial growth factor (VEGF), and (**D**) fibroblast growth factor-2 (FGF2). The results were normalized to the mRNA expression level of ß-actin and shown relative to cells transfected with siCtrl. Data are represented as the mean ± the standard deviation for a minimum of three independent experiments. (ns = not significant *p* > 0.05, * *p* ≤ 0.05, ** *p* ≤ 0.01, *** *p* ≤ 0.001, **** *p* ≤ 0.0001).

To demonstrate the therapeutic potential of our nanoformulations for promoting the upregulation of angiogenesis factors, siRNA targeting prolyl hydroxylase domain protein 2 (siPHD-2) or non-specific control siRNA (siCtrl) was formulated (30 nM) in both our nanoformulations and the two commercial transfection reagents (Lipofectamine^®^ RNAiMAX and jetPRIME^®^). To increase the chances for effective PHD-2 downregulation, we used a siPHD-2 pool containing three sequences (Table S2). NIH-3T3 cells were transfected with carriers for 4 h, after which cells were washed and incubated further with cell culture medium without or with 20 μM DES. After 20 h, cells were washed again and supplemented with fresh cell culture medium for an additional 24 h. After that, RNA was extracted and gene silencing was evaluated by qRT-PCR. In the absence of DES, AuNP@PLA, Lipofectamine^®^ RNAiMAX and jetPRIME^®^ induced significant PHD-2 mRNA silencing in contrast to AuNP@CS (Figure 5A). Treatment of cells with DES only had a significant effect on the knockdown efficiency of AuNP@PLA, in line with our observations on endosomal escape. AuNP@PLA combined with DES treatment resulted in the highest knockdown of 87%. As mentioned before, PHD-2 activity triggers hydroxylation and degradation of the pro-angiogenic transcription factor hypoxia-inducible factor 1α (HIF-1α). When PHD-2 is naturally inactivated (i.e., under hypoxic conditions), or, in our case, by silencing through RNAi, its absence prevents HIF-1α protein catabolism. To confirm that this is the case, we investigated the efficacy of siPHD-2 on HIF-1α transcriptional activity. For all carriers, a significant increase in HIF-1α transcriptional activity (Figure 5B) was indeed observed, proportional to their level of PHD-2 downregulation. Again, only in the case of AuNP@PLA, DES treatment had a significant effect on HIF-1α expression.

When the intracellular concentration of HIF-1α increases, it can translocate to the nucleus and dimerize with HIF-1β. This, in turn, induces expression of angiogenesis factors such as vascular endothelial growth factor (VEGF), fibroblast growth factor 2 (FGF-2), and others. In agreement with the above mentioned PHD-2 suppressive effect of AuNP@PLA with DES treatment, the mRNA level of VEGF and FGF-2 from cells treated with this approach was indeed higher than that from cells treated with other carriers (Figure 5C,D).

### 2.6. Enhanced HIF-1α Expression Stimulates Migration and Proliferation In Vitro

**Figure 6:**
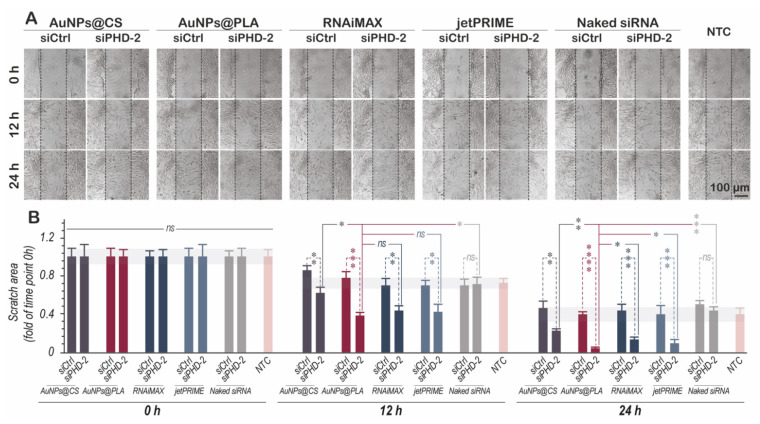
**Effects of siPHD-2 transfections on the migration and proliferation of NIH-3T3 cells in a wound scratch assay.** (**A**) Representative microscopy images of scratch assays. NIH-3T3 cells were treated with siPHD-2- or siCtrl-loaded nanoformulation for 4 h, followed by 20 h incubation with cell culture medium supplemented with 20 μM DES. After washing and adding fresh cell culture medium, scratches were made of about 100 µm wide. Microscopy images were taken immediately after (0 h) as well as after 12 h and 24 h. The black dashed lines indicate the initial scratch boundaries. The scale bar corresponds to 100 μm. (**B**) Quantitative analysis of the migration area was performed using Image J software at 0 h, 12 h and 24 h. The data are represented as the mean ± standard deviation. (ns = not significant *p* > 0.05, * *p* ≤ 0.05, ** *p* ≤ 0.01, *** *p* ≤ 0.001, **** *p* ≤ 0.0001).

The goal of wound healing is re-epithelialization, or wound closure, which is the most important part of the healing process. This process is facilitated by the migration of cells at the margin towards the center of the wound [81]. A key feature of fibroblasts is that they provide the contractile forces to bring the wound edges together, for which their migratory capacity is crucial [82]. For studying migration and proliferation, an in vitro wound-healing scratch assay with mouse NIH-3T3 fibroblasts was carried out [83]. Figure 6A shows representative microscopy images of NIH-3T3 cells treated with the various nanoformulations (30 nM siPHD-2) and 20 µM DES from which the scratch area (=area not covered by cells) was quantified over time. At time 0 h, all scratch areas were identical (Figure 6B). However, differences became clearly visible after 12 h and 24 h. For all nanoformulations, siPHD-2 had a significant positive effect on wound healing compared to siCtrl or naked siPHD-2. AuNP@PLA again performed better than AuNP@CS and similar to the commercial transfection reagents RNAiMAX and jetPRIME^®^. After 24 h, the gaps were completely covered again for cells treated with AuNP@PLA, RNAiMAX and jetPRIME^®^. Overall, these data show that silencing of PHD-2 accelerated wound healing by enhancing the migration of fibroblasts.

## 3. Discussion

Diabetic wounds suffer from impaired neovascularization [25,26]. Although the extracellular environment of diabetic wounds is hypoxic, which normally increases the stability and expression of HIF-1α, studies have shown that HIF-1α levels are reduced in diabetic wounds [42]. In addition, it is no longer able to bind to its dimer (HIF-1β) in the nucleus, which hinders the transcriptional activation of downstream genes such as VEGF and FGF, which play a critical role in the neovascularization and angiogenesis response. By virtue of HIF-1α’s ability to upregulate multiple genes encoding critical angiogenic growth factors, HIF-1α has become an attractive molecular target for the treatment of diabetic wound healing. We opted for a RNAi-based approach for silencing of PHD-2, which is known to induce natural degradation of HIF-1α, so as to upregulate angiogenesis factor genes and increase neovascularization. As siRNA delivery requires that the nucleic acid should be protected from degradation on its way to the target cells and must overcome intracellular barriers in order to be effective, we designed highly stable layer-by-layer carriers with AuNPs at its core. In particular, we explored the effect of the outer polymeric layer (chitosan and poly L-arginine) on delivery efficacy, toxicity and subsequent biological effect in NIH-3T3 fibroblasts. We also studied the synergistic effect with the cationic amphiphilic drug desloratadine to increase transfection efficiency by boosting the nanocarrier’s endosomal escape efficiency.

### 3.1. Nanoformulation Characterization

Through a previously published LbL approach [64], two gold nanoparticle-based formulations were successfully synthesized, which only differed in the final polymeric layer (CS or PLA). UV–vis, DLS and gel electrophoresis confirmed successful siRNA complexation and coating with the final polymer layer. The type of outer polymer layer did not have a significant effect on the nanoformulation’s loading capacity, size, zeta potential, stability, or siRNA release profile (Figure 1 and Figure S1).

### 3.2. Nanoformulation Cytotoxicity and Uptake

Biocompatibility of nanocarriers is a crucial feature. We found that toxicity was acceptable in NIH-3T3 cells (70% viability) when AuNP@CS and AuNP@PLA were added at a concentration equivalent to 30 nM siRNA. Moreover, when combined with 20 µM DES, toxicity did not markedly increase.

Cellular internalization is facilitated by electrostatic interaction between positive carriers and the negative cell membrane. Consequently, the outer layer of the NPs plays a key role to facilitate interaction with the cell membrane as a first step towards efficient internalization by endocytosis. Analysis of cellular uptake revealed that a PLA outer layer was beneficial for efficient internalization as compared to CS. This may be due to guanidinium groups present in PLA, which can specifically interact with the sulfate groups of glycosaminoglycans present on cell membranes, and consequently, facilitate the uptake of NPs [84].

### 3.3. Endosomal Escape Efficiency in Combination with DES Treatment

Chitosan is a cationic polysaccharide with pKa around 6.3 that can form complexes with negatively charged nucleic acids. At pH values below its pKa (i.e., endosomal pH), the primary amine groups on chitosan become protonated [85], leading to endosomal buffering, osmotic swelling and subsequent endosome disruption [65] (Scheme 2). Poly L-arginine (PLA) is a homopolymer containing a high percentage of cationic L-arginine amino acids. Structurally, PLA is the simplest cell penetration peptide (CPP) mimic, with arginine as the only building block, whose membrane permeability mainly relies on their guanidinium charged groups [69]. As PLA has a pKa > 12, it is expected to be fully protonated when they are incorporated into endosomes, ruling out any possible proton sponge effect. Instead, it can bind to the lipid bilayers, leading to internal stress that can be sufficiently strong to create pores in the endosomal lipid membranes [86] (Scheme 2).

As the outer layer on the LbL-NPs will define the interaction with the cell membrane and the endosomes, the release mechanism of AuNP@PLA and AuNP@CS is expected to differ. In a previous study, it was observed that LbL-AuNPs with CS as the outer layer had high endosomal escape efficiency in a H1299 lung carcinoma cell line [64]. However, in our study, the chitosan polymer in AuNP@CS did not show good ability to escape from the endosomes in NIH-3T3 cells. One possible explanation for this observation could be linked to the low percentage of cellular uptake of these nanoparticles (Figure 2C,D) in this cell type. As such, it is conceivable that the amount of polymer per endosome does not allow sufficient osmotic pressure to be created in order to rupture them. Consequently, the nanoparticles and their siRNA cargo will remain trapped inside endolysosomes (Figure 4A). On the other hand, AuNP@PLA alone (i.e., without the presence of DES) already showed 25% of cells with one or more endosomal escape event (Figure 4B).

Despite clear DES-induced lysosomal swelling in combination with all NPs tested (Figure 3A,B and Figure 4A), it only enhanced the endosomal escape and transfection efficiency of AuNP@PLA, but not of AuNP@CS, Lipofectamine^®^ RNAiMAX, or jetPRIME^®^. As reported previously by Van de Vyver et al., DES can induce pores in the membrane of endolysosomes, allowing efflux of compounds with a molecular weight up to ~150 kDa [61]. The molecular weight of siRNA falls below this threshold (~14 kDa), so that DES-induced pores should be large enough to allow siRNA to pass through [61]—that is, if siRNA is released from the carriers in the endolysosomal compartment. In a forced siRNA release assay, in which siRNA is displaced from the carriers by adding SDS, it was found that siRNA less easily dissociates from AuNP@CS and jetPRIME^®^. Therefore, a potential explanation as to why DES did not increase the transfection efficiency of AuNP@CS and jetPRIME is because siRNA is insufficiently released from those nanocarriers. Instead, in the SDS assay, it was found that siRNA dissociated more easily from AuNP@PLA, which can explain why DES did have an added effect on its transfection efficiency. However, siRNA was found to dissociated as easily from Lipofectamine^®^ RNAiMAX as from AuNP@PLA, so that likely, a different reason exists why RNAiMAX did not show enhanced endosomal release in combination with DES [61,87]. Lipid carriers such as Lipofectamine^®^ RNAiMAX immediately release the encapsulated siRNA in the cytosol due to fusion with the endosomal membrane and possibly even already at the level of the plasma membrane. Thus, it is not expected for Lipofectamine^®^ RNAiMAX to lead to a lysosomal depot of siRNA, which is available for CAD-enhanced endosomal escape. Regarding the absence of a beneficial effect of DES treatment in combination with AuNP@CS or jetPRIME^®^, we would like to note that this could also be related to the fact that the effect of DES relies on an acidic pH for its accumulation in the endolysosomes [61]. Considering that CS and PEI have buffering capacity, it may be that DES accumulates less in endolysosomes containing AuNP@CS or jetPRIME^®^. Finally, regarding the additive effect observed of DES in combination with AuNP@PLA, this could be additionally related to intralysosomal degradation of PLA, leading to an increased concentration of free L-arginine inside the endolysosomes. As it is known that the presence of this amino acid can induce the formation of pores in lipid bilayers, this may have contributed to the additive effect for endosomal escape in combination with DES [66,67,68,69].

### 3.4. Effect of PHD-2 Silencing on the Expression of Angiogenesis Factors

Post-transcriptional gene silencing via RNAi has great potential for the treatment of a variety of disorders [52,53]. In the particular context of wound healing, the stabilization of HIF-1α by selective silencing of PHD-2 is an attractive approach for angiogenic therapy and neovascularization in a diabetic wound bed that is characterized by impaired vascularity and a deficient hypoxic response [25,26,27]. To this end, we tested the efficacy of PHD-2 knockdown as well as the ability to affect the expression of multiple angiogenic growth factors downstream through the stabilization of HIF-1α, in NIH-3T3 fibroblasts. Our results showed that a significant reduction in PHD-2 mRNA levels was achieved after delivery of siPHD-2 by all carriers evaluated in this study (Figure 5A). In addition, PHD-2 silencing was proven to protect HIF-1α from proteasomal degradation (Figure 5B) and induced a significant increase in both angiogenic growth factors VEGF and FGF2 (Figure 5C,D). These results were strongly correlated with the percentage of endosomal escape obtained based on confocal microscopy.

DES treatment of cells proved to be most effective when combined with AuNP@PLA. This led to higher expression of VEGF and FGF-2 angiogenesis factors, even to a higher extent than the commercial carriers Lipofectamine^®^ RNAiMAX and jetPRIME^®^. Our results also showed that PHD-2 silencing enhances the migration and proliferation of NIH-3T3 cells evaluated by a scratch assay. This observation could be attributed to the increased expression of growth factors (FGF-2, VEGF) that are known to induce signal-promoting cell proliferation and faster wound healing.

## 4. Materials and Methods

### 4.1. Materials

HAuCl4, chitosan (CS, low molecular weight, degree of deacetylation: 80%), and poly L-arginine hydrochloride (PLA, molecular weight > 70,000) were purchased from Sigma-Aldrich. Dulbecco’s Modified Eagle Medium (DMEM), L-Glutamine, Penicillin/Streptomycin solution (5000 IU/mL penicillin and 5000 μg/mL streptomycin) (P/S), Calf Bovine Serum (CBS), Trypan Blue, 0.25% Trypsin-EDTA, and Dulbecco’s phosphate-buffered saline (DPBS) were supplied by Gibco BRL (Merelbeke, Belgium). Lipofectamine^®^ RNAiMAX reagent was purchased from Invitrogen. jetPRIME^®^ was purchased from Polyplus-transfection^®^ company (Illkirch, France). CellTrace™ Yellow and Hoechst 33342 were purchased from Molecular Probes™ (Erembodegem, Belgium). CellTiter-Glo^®^ Luminescent Cell Viability Assay was purchased from Promega (Leiden, Netherlands). siRNA against PHD-2 (siPHD-2) (Table S2), and negative ctrl siRNA (siCtrl) was purchased from IDT (Integrated DNA Technologies, Leuven, Belgium). For uptake experiments, TYE 563 Transfection Control DsiRNA (TYE563 siRNA) was used (Integrated DNA Technologies, Leuven, Belgium). For endosomal escape, AlexaFluor647-labeled oligonucleotides (AF647 ONs) were used (Eurogentec). Lysosomes were labeled with LysoTracker^®^ Deep Red (LDR) and LysoSensor™ Green DND-189 (Molecular Probes™, Ghent, Belgium).

### 4.2. Nanoformulations Synthesis

Layer-by-layer gold nanoparticles (LbL-AuNP@CS/PLA).

First layer (including gold core): Gold nanoparticles capped with chitosan (AuNPs) were synthesized by the reduction of HAuCl4 directly by chitosan as previously reported [64]. Briefly, 85 μL of 25 mM HAuCl4 was added to preheated chitosan (200 mL of 0.5% (*w*/*v*) chitosan solution dissolved in 1% (*v*/*v*) acetic acid) drop by drop under continuous magnetic stirring and reflux for 1h until the color turned deep red. Finally, the synthesized NPs were centrifuged at 22,000× *g*, 4 °C, for 1 h and dispersed in deionized water for further use.

Second layer: To load negatively charged siRNA molecules onto the chitosan-coated AuNPs as a second layer, AuNPs were resuspended in 10 mM HEPES buffer with pH = 7 and mixed with siRNA at 1:10 weight ratios of siRNA to Au atoms, under continuous stirring for 1 h.

Third layer: Lastly, a final chitosan (AuNP@CS) or poly L-arginine (AuNP@PLA) layer was applied by adding the nanoparticles to a 0.5% (*w*/*v*) CS or PLA solution, followed by continuous stirring for 1 h. Excess of chitosan or PLA was removed by centrifugation at 22,000× *g*, 4 °C, 1 h, and the purified particles were resuspended and stored in RNase-free water.

### 4.3. Characterization of Nanoformulations

− UV–vis absorbance spectroscopy

UV–Visible spectroscopy was used to characterize spectral changes in the localized surface plasmon resonance (LSPR) band of AuNPs after the deposition of each layer. Spectra were recorded in the range of 200–900 nm using a Thermo Scientific NanoDropTM spectrophotometer.

− Dynamic light scattering (DLS) measurements

The hydrodynamic size and zeta potential of the particles were measured using a Malvern Zetasizer Nano ZS instrument (Malvern, UK) with a He/Ne laser (633 nm).

− Fourier transform infrared (FTIR) spectroscopy

To confirm the presence of chitosan and poly L-arginine on AuNPs, their FTIR spectra were acquired. For this, AuNP@CS or AuNP@PLA were lyophilized by placing the samples on a temperature-controlled steel shelve. The shelve temperature was subsequently decreased to −40 °C at 1 °C/min. Next, the drying chamber was made into a vacuum (100 µbar). Subsequently, the shelve temperature was increased to −25 °C for primary drying and was kept constant for 24 h. Finally, secondary drying was achieved by increasing the shelve temperature to 20 °C at 0.1 °C/min. After 10 h of secondary drying, the chamber was vented with dry nitrogen gas. The product was pressed against the crystal. Spectra were collected with an ATR-FTIR Nicolet IS5 spectrophotometer (Thermo Fisher, Waltham, MA, USA) over a range of 4000 to 500 cm^−1^ with a resolution of 4 cm^−1^ on ATR-diamond crystal.

#### Gel Electrophoresis Assay

After the addition of each layer, NPs were centrifugated at 22,000× *g*, 4 °C, 1 h and the supernatants and dispersed pellets were evaluated for siRNA complexation with gel electrophoresis. A 1% agarose gel containing 1:10,000 of Gel-REDTM stain (Biotium, Hayward, CA, USA) was prepared by dissolving agarose (UltraPure Agarose, Invitrogen, Erembodegem, Belgium) in TBE buffer (98 mM Tris, 88 mM Boric acid, 2 mM Na2EDTA with pH 8). Next, 20 μL of supernatant or dispersed NP pellets in water or 2% SDS solution was loaded onto gel. Gel electrophoresis was carried out in a horizontal gel electrophoresis unit (Bio-Rad Laboratories, Richmond, CA, USA) at 100 V for 30 min in TBE buffer. Fluorescence bands were visualized using a Kodak digital science camera (Kodak EDAS 120, Rochester, NY, USA) under UV light excitation (Bio-Rad UV transilluminator 2000, Richmond, CA, USA).

### 4.4. siRNA Release from the Nanoformulations

To evaluate the release profile of siRNA, we used the separation and analysis method as previously reported [64]. Briefly, AuNP@CS or AuNP@PLA were suspended in 10 mM HEPES buffer at pH 7.4, distributed over multiple microtubes and gently shaken for up to 7 days at 37 °C. At specific time intervals, one microtube was taken and centrifuged at 22,000× *g* for 1 h at 4 °C. The concentration of siRNA in the supernatant was measured using a Thermo Scientific NanoDropTM spectrophotometer at 260 nm to determine the percentage of released siRNA.

### 4.5. Lipofectamine^®^ RNAiMAX and jetPRIME^®^ Preparation

Lipofectamine^®^ RNAiMAX and jetPRIME^®^ were used to benchmark the performance of the LbL-AuNPs. siRNA complexes with those transfection agents were prepared freshly at the time of transfection according to the manufacturer’s protocol. For RNAiMAX, 15 pmol of siRNA was transferred in 25 μL Opti-MEM and, in a separate tube, 1.5 μL Lipofectamine^®^ RNAiMAX reagent was added to 25 μL Opti-MEM. Next, both solutions were mixed (1:1 ratio) and incubated for 5 min at room temperature. Finally, the formed complexes were diluted in Opti-MEM to reach the proper siRNA concentration, which was added to the cells. For jetPRIME^®^, 27.5 pmol of siRNA was diluted in 50 μL jetPRIME^®^ buffer, vortexed for 10 s and spun down with a mini centrifuge. Then, 2 μL of jetPRIME^®^ reagent was added, vortexed for 1 s, spun down and incubated 10 min at room temperature. Finally, the formed complexes were diluted in DMEM supplemented with serum and added to the cells at the desired concentration.

### 4.6. Dissociation Degree of Nanoformulations by Gel Electrophoresis and Fluorescence Fluctuation Spectroscopy (FFS)

Nanoformulations loaded with siRNA labeled with TYE 563 dye (TYE563 siRNA) were prepared as described above. Next, the release of siRNA from the nanoformulations was evaluated upon addition of the poly *anionic* surfactant sodium dodecyl sulfate (SDS) in different concentrations. For this, equal volumes of SDS and siRNA-loaded formulations in HEPES buffer were mixed and incubated at room temperature for 10 min.

For gel electrophoresis, 20 μL of this solution was mixed with 5 μL of 5× gel loading buffer, after which 20 µL of the mixture was loaded onto the gel, which was run for 30 min at 100 V before imaging.

Fluorescence fluctuation spectroscopy (FFS) is a fluorescence microscopy-based technique that measures the continuous movement of fluorescent molecules diffusing in and out of the detection volume of a confocal microscope [89,90,91]. Previous work by our group used FFS to quantify the association and dissociation of fluorescently labeled molecules in various nanocarriers [91,92,93,94].

For FFS measurements, the solution of nanoformulations with SDS was transferred to a glass-bottom 96-well plate (Greiner Bio-One GmbH, Frickenhausen, Germany). The focal volume of the microscope was adjusted in the sample, followed by the recording of the fluorescence fluctuations for 60 s. FFS experiments were performed on a laser scanning confocal microscope (C1si, Nikon, Japan) equipped with a water immersion objective lens (60× Plan Apo VC, N.A. 1.2, Nikon, Japan), using a 633 nm laser line for the excitation of fluorescent siRNA (TYE563 siRNA), and the fluorescence signal was recorded with the detection channels of the fluorescence correlation spectrometer MicroTime 200 (Picoquant GmbH, Berlin, Germany) controlled by SymPhoTime software (Picoquant GmbH, Germany). All samples were prepared in triplicate. The average fluorescence intensity of freely diffusing and complexed siRNA in the fluorescence fluctuation profile was determined as described previously [92,95], from which the percentage of released or complexed siRNA can be derived.

### 4.7. Cell Culture

NIH-3T3 mouse embryonic fibroblast cells (ATCC^®^ CRL-1658™) were used as a fibroblast cell model in this study. The passage number was always kept below 20. NIH-3T3 cells were cultured in Dulbecco’s Modified Eagle Medium (DMEM) supplemented with 10% calf bovine serum (CBS), 4 mM L-glutamine, 1 mM sodium pyruvate, 1500 mg/L sodium bicarbonate and 1% pen-strep at 37 °C in a 5% CO_2_ humidified environment. To model diabetic hyperglycemia, NIH-3T3 fibroblasts were cultured in DMEM supplemented with approximately 4500 mg/L or 25 mM D-glucose, which approximates diabetic levels of glucose in vivo. Culture medium was renewed every other day, unless the 80% confluence level was reached, in which case, the cells were split using 0.25% trypsin-ethylenediaminetetraacetic acid (EDTA).

### 4.8. Cell Viability Assay

Evaluation of cytotoxicity was performed by the CellTiter-Glo^®^ luminescent Cell Viability Assay (Promega, Belgium) according to the manufacturer’s instructions. Briefly, 100 µL of a suspension of 80,000 cells/mL were added in individual wells of 96-well flat-bottomed culture plates. After 24 h, different nanoformulations were diluted in complete DMEM (100 µL) at different effective siRNA concentrations (5–40 nM) and incubated for 4 h at 37 °C with the cells. Next, the cells were washed and incubated for another 20 h with fresh DMEM, with or without DES, depending on the experiment. Finally, DMEM was removed and replaced with 100 µL of pre-heated CellTiter-Glo^®^ reagent and 100 µL of fresh DMEM, and shaken for 10 min at 120 RPM at room temperature to induce complete cell lysis and to allow the signal to stabilize. Next, 100 µL of each well was transferred to white opaque 96-well plates and the luminescence signal was recorded by a GloMax™ 96 microplate luminometer (Promega, Belgium). Data were presented as the mean cell viability (percentage of luminescent signal relative to non-treated cells (NTC) for each condition) ± standard deviation (SD) for minimum three independent repeats.

### 4.9. Quantification of Nanoformulation Internalization by Flow Cytometry

To quantify the cellular uptake of siRNA-loaded formulations by flow cytometry, NIH-3T3 cells were seeded in 96-well plates at a density of 10,000 cells/well and allowed to attach overnight. The next day, carriers loaded with TYE563 siRNA were incubated with the cells for 4 h at different concentrations (37 °C, 5% CO_2_). Next, cells were washed with DPBS and detached from the well plates using trypsin/EDTA 0.25%, diluted with DMEM, transferred to U-Bottom 96-well plates, centrifuged at 500× *g* for 5 min, and finally, cell pellets were resuspended in flow buffer (DPBS supplemented with 0.1% sodium azide and 1% BSA). Red fluorescence (638 nm excitation with laser and detection with a 660/20 nm bandpass filter) was measured for a minimum of 10,000 cells using the CytoFLEX flow cytometer. For calculating rMFI, the following equation was used:

$$\mathrm{rMFI}\;\left(\mathrm{relative}\;\mathrm{Mean}\;\mathrm{Fluorescence}\;\mathrm{Intensity}\right)=\frac{\mathrm{MFI}\;\mathrm{of}\;\mathrm{cells}\;\mathrm{treated}\;\mathrm{with}\;\mathrm{TYE}563\;\mathrm{siRNA}}{\mathrm{MFI}\;\mathrm{of}\;\mathrm{cells}\;\mathrm{treated}\;\mathrm{with}\;\mathrm{nonlabeled}\;\mathrm{siRNA}}$$

### 4.10. Visualizing Nanoformulation Internalization by Confocal Microscopy

To visualize the cellular uptake of siRNA by confocal microscopy, NIH-3T3 cells were seeded in 35 mm CELLview glass bottom microscopy dishes (Greiner Bio-One, Vilvoorde, Belgium) at a density of 100,000 cells/mL and left to settle overnight. The next day, nanoformulations loaded with TYE563 siRNA were incubated with the cells for 4 h at 10 nM or 30 nM of siRNA effective concentration (37 °C, 5% CO_2_). Next, cells were washed with DPBS, and the cell’s cytoplasm was stained by incubation with CellTrace™ Yellow (Molecular Probes™, Ghent, Belgium) in DPBS (5 mM in DMSO, 1/1000 dilution) for 20 min at 37 °C. After this, cells were washed 2 times with DPBS and incubated with Hoechst 33,342 (Molecular Probes™, Ghent, Belgium) in DPBS (1 mg/mL in water, 1/1000 dilution) for 15 min at 37 °C. After staining, cells were washed with DPBS, supplemented with fresh DMEM and kept at 37 °C in a humidified atmosphere containing 5% CO_2_ until confocal imaging.

A Nikon A1R HD confocal (Nikon, Japan), equipped with a laser box (LU-N4 LASER UNIT 405/488/561/640, Nikon Benelux, Brussels Belgium), detectors (A1-DUG-2 GaAsP Multi Detector Unit, GaAsp PMT for 488 and 561 and Multi-Alkali PMT for 647 and 405 nm), and a 20× air objective lens (CFI plan Apo VC 20×, NA 0.75, WD 1000 µm) (Nikon, Japan) were used for imaging. Images were acquired using the NIS Elements software (Nikon, Japan). The 408 nm, 638 nm and 561 nm laser lines were applied to excite the Hoechst-labeled nuclei, the fluorescence resulting from TYE 563 siRNA and CellTrace™ Yellow-labeled cytoplasm, respectively.

### 4.11. Quantification of Lysosomal Volume by Flow Cytometry

NIH-3T3 cells were seeded in 96-well plates at a density of 8000 cells/well (100 μL/well) and were allowed to settle overnight. On the next day, the cells were incubated with siCtrl-loaded carriers for 4 h at 37 °C in a humidified atmosphere with 5% CO_2_. Subsequently, cells were washed and incubated with fresh DMEM without or with 10 or 20 μM DES for 20 h. Afterwards, (endo)lysosomes were labeled with LysoTracker^®^ Deep Red (LDR) (Molecular Probes™, Ghent, Belgium) through incubation with 50 μL 75 nM LDR in DMEM for 30 min at 37 °C. After washing with DPBS, further sample preparations for flow cytometry were carried out as described above. For each sample, the side scatter (SSC) as well as the red fluorescent signal was measured by a 660/20 emission filter, which was excited with 638 nm laser line. The fold changes in LDR signal intensity/SSC signal are presented as the mean ± standard deviation (SD) for a minimum of three independent repeats (biological replicates).

### 4.12. Visualization of Lysosomes by Confocal Microscopy

For visualization of lysosomes by confocal microscopy, NIH-3T3 cells were seeded in 35 mm CELLview glass bottom microscopy dishes, incubated with nanoformulations loaded with TYE 563 siRNA and treated with DES similarly as described for the quantification of lysosomal volume. Following 20 h of DES treatment, DMEM was removed and replaced with fresh medium containing LysoSensorTM Green DND-189 (Molecular Probes™, Ghent, Belgium) (1 μM) for 30 min at 37 °C. After 30-min incubation, cell nuclei were stained with Hoechst 33342 (Molecular Probes™, Ghent, Belgium) in DPBS (1 mg/mL in water, 1/1000 dilution) for 15 min at 37 °C and washed 2 times with DPBS. Finally, fresh DMEM was added, and cells were kept at 37 °C in a humidified atmosphere containing 5% CO2 until confocal imaging. Imaging was carried out with a 60× oil objective lens (CFI plan Apo VC 60×, NA 1.40, WD 0.13) (Nikon, Japan) on a Nikon A1R HD confocal microscope (Nikon, Japan). Images were recorded using the NIS Elements software (Nikon, Japan). The 408 nm, 638 nm and 488 nm laser lines were applied to excite the Hoechst-labeled nuclei, the fluorescence resulting from TYE563 siRNA and the LysoSensorTM Green DND-189, respectively.

### 4.13. Visualization and Quantification of the Cytosolic Release of AF647 ONs

Visualization and quantification of endosomal escape was performed based on a dequenching assay published before [80,96]. To assess endosomal escape, nanoformulations were loaded with AlexaFluor647-labeled 21mer oligonucleotides (AF647 ONs) instead of siRNA. Upon endosomal escape, the labeled ONs will be released into the cytoplasm and finally accumulate in the nucleus. A red fluorescent nucleus is then a sign that at least one endosomal escape event happened in a particular cell. For this experiment, NIH-3T3 cells were seeded in 35 mm CELLview glass bottom microscopy dishes (Greiner Bio-One, Vilvoorde, Belgium) at a density of 80,000 cells/mL and left to settle overnight. On the next day, cells were washed and incubated with the LbL-AuNPs or with commercial transfection agents (Lipofectamine^®^ RNAiMAX and jetPRIME^®^) all containing AF647 ONs. Naked AF647 ONs without carriers were used as a control. Following an incubation of 4 h (37 °C, 5% CO_2_), the dispersion was removed and cells were washed once with PBS (Invitrogen, Merelbeke, Belgium). Next, cells were incubated with 1.5 mL of fresh DMEM without or with 20 µM of DES for 20 h (37 °C, 5% CO_2_). On the next day, after removing the medium, cell nuclei were stained with Hoechst 33342 (Molecular Probes™, Ghent, Belgium) in DPBS (1 mg/mL in water, 1/1000 dilution) for 15 min at 37 °C and washed 2 times with DPBS. Finally, fresh DMEM was added, and cells were kept at 37 °C in a humidified atmosphere containing 5% CO_2_ until confocal imaging. Images were obtained with a 60× oil objective lens (CFI plan Apo VC 60×, NA 1.40, WD 0.13) (Nikon, Japan) on a Nikon A1R HD confocal microscope (Nikon, Japan). Images were acquired using the NIS Elements software (Nikon, Japan). The 408 nm and 638 nm laser lines were used to excite the Hoechst-labeled nuclei and the AF647 ONs, respectively. During data analysis with ImageJ (FIJI) software [97], nuclei were detected in the blue channel by thresholding (applying the same offset values for every image), subsequently allowing determination of the intensity (mean gray value) of the nuclear AF647 ON fluorescence signal in the red channel. From this, the percentage of cells with a AF647 ON-positive nucleus was determined. Data are represented as the percentage of cells with AF647 ON positive nuclei, as determined from at least 500 cells in a minimum of 25 images.

### 4.14. Transfection Efficiency Analysis by Quantitative Reverse Transcription Polymerase Chain Reaction (qRT-PCR)

To evaluate siRNA knockdown of PHD-2 and its effect on other genes, real-time quantitative reverse transcription polymerase chain reaction (qRT-PCR) was performed. NIH-3T3 cells were seeded in 12-well plates at a density of 80,000 cells/mL and were allowed to settle overnight. On the next day, the cells were incubated with siRNA-loaded nanoformulations for 4 h at 37 °C in a humidified atmosphere containing 5% CO_2_. Note that for every siPHD-2 condition, a siCtrl sample was included to account for potential off-target effects. Subsequently, the carrier dispersion was removed, and the cells were incubated with DMEM without or with 20 μM DES for 20 h. Afterward, the medium was removed and cells were kept in fresh DMEM for an additional 24 h and washed with DPBS before RNA extraction. The total cellular RNA was extracted with the Aurum™ Total RNA Mini Kit (Bio-Rad, Hercules, CA, USA) according to the manufacturer’s instructions. After quantification of RNA with a Thermo Scientific NanoDropTM spectrophotometer, 100 ng of RNA was reverse transcribed to cDNA using the iScript cDNA synthesis kit (Biorad, Hercules, CA, USA) according to the manufacturer’s protocol. Quantitative real-time reverse transcription PCR (qRT-PCR) was performed using iTaq™ Universal SYBR^®^ Green Supermix (Bio-Rad, Hercules, CA, USA) on a CFX384 Touch Deep Well Real-Time PCR Detection System (Bio-Rad, Hercules, CA, USA). The reaction conditions consisted of 10 μL reaction volumes with 4.5 μL diluted cDNA template in PCR-grade water, 5 μL iTaq™ Universal SYBR^®^ Green Supermix, and 0.5 μL of each primer (100 nM). The sequences of the forward and reverse primers are listed in Table S3. The amplification procedure was carried out as follows: initial denaturation at 95 °C for 30 s, followed by 40 cycles of 95 °C for 5 s and 56.5–60 °C (based on annealing/extension of each primer) for 30 s. The comparative cycle threshold (Ct) method was used, and the relative quantification of the target gene was normalized to that of the β-actin expression level using the 2^−ΔΔCt^ method in the CFX Maestro™ Software.

### 4.15. In Vitro Scratch Wound and Cell Migration Assays

The effect of siPHD-2 knockdown on cell migration and wound closure was investigated by the scratch cell migration assay. NIH-3T3 cells were seeded in 6-well plates at a density of 80,000 cells/mL and were allowed to settle overnight. On the next day, the cells were incubated with siRNA-loaded nanoformulations for 4 h at 37 °C in a humidified atmosphere containing 5% CO_2_. Subsequently, the nanoformulations’ dispersion was removed, and the cells were incubated with DMEM containing 20 μM DES for 20 h. The next day, a scratch was made using a sterile 200 μL pipette tip, which was scraped across each well, creating a cell-free area. The cells were then rinsed with PBS to remove any free-floating cells and debris. DMEM was then added, and culture plates were incubated at 37 °C for 24 h. Images of scratched areas were captured with an inverted microscope (Nikon Eclipse TS100, Japan) immediately, 12 h and 24 h after scratching. The scratch area (=area not covered by cells) was measured using the ImageJ software. The scratch area was expressed as the scratch closure in relation with the initial scratched area: A1/A0, where A0 is the scratched area at time 0 and A1 is the corresponding scratched area at 12 or 24 h

### 4.16. Statistical Analysis

All experiments were performed in triplicate. All the results are reported as mean ± standard deviation (SD). Statistical analyses were performed using one-way ANOVA to compare multiple conditions and Student’s *t*-test for direct comparison of 2 conditions; a *p*-value < 0.05 was considered statistically significant (* *p* ≤ 0.05, ** *p* ≤ 0.01, *** *p* ≤ 0.001, **** *p* ≤ 0.0001).

## 5. Conclusions

In this study, we demonstrate that siRNA can be formulated into a tunable layer-by-layer platform around gold core particles, for which the outer layer can be conveniently modified, which allows tuning of cellular internalization and endosomal escape. Of the two different outer layers tested, it was found that PLA (AuNP@PLA) not only has an outstanding stability over time as an siRNA carrier, but also proved to be highly effective for cytosolic release after endocytic uptake. Moreover, AuNP@PLA combined with DES treatment of cells resulted in a boosting of cytosolic release of siRNA, while this was not the case for AuNP@CS or any of the tested commercial transfection reagents. In NIH-3T3 fibroblast cells, we found that siRNA-mediated downregulation of PHD-2 resulted in increased levels of VEGF and FGF-2 angiogenesis factors. These in vitro results could pave the way for a novel nanoparticle-based angiogenic siRNA therapy for improved healing of diabetic wounds.

## Data Availability

The data presented in this study are available on request from the corresponding author.

## Supplementary Materials

The following are available online at https://www.mdpi.com/article/10.3390/ijms22179216/s1.
