# Supplementary material for: Increasing Angiogenesis Factors in Hypoxic Diabetic Wound Conditions by siRNA Delivery: Additive Effect of LbL-Gold Nanocarriers and Desloratadine-Induced Lysosomal Escape
Source: Int J Mol Sci. 2021 Aug 26;22(17):9216. doi: 10.3390/ijms22179216 (PMC8431033; doi:10.3390/ijms22179216)
Supplement: Supplementary file 1 [file ijms-22-09216-s001.zip › ijms-1342784-supplementary.pdf]

International Journal of *Molecular Sciences*

MDPI

Article

Increasing angiogenesis factors in hypoxic diabetic wound conditions by siRNA delivery: additive effect of LbL-gold nanocarriers and desloratadine-induced lysosomal escape

Elnaz Shaabani [1,2], Maryam Sharifiaghdam [1,2], Joris Lammens [3], Herlinde De Keersmaecker [1,4], Chris Vervaet [3], Thomas De Beer [5], Elahe Motevaseli [6], Mohammad Hossein Ghahremani [7], Parvin Mansouri [8], Stefaan De Smedt [1,4], Koen Raemdonck [1], Reza Faridi-Majidi [2,*], Kevin Braeckmans [1,4,*] and Juan C. Fraire [1]

1 Laboratory of General Biochemistry and Physical Pharmacy, Faculty of Pharmacy, Ghent University, Ottergemsesteenweg 460, 9000 Ghent, Belgium; elnaz.shaabanisichani@ugent.be (E.S.); maryam.sharifiaghdam@ugent.be (M.S.); herlinde.dekeersmaecker@ugent.be (H.D.K.); Stefaan.Desmedt@ugent.be (S.D.S.); koen.raemdonck@UGent.be (K.R.); juan.fraire@ugent.be (J.C.F.)
2 Department of Medical Nanotechnology, School of Advanced Technologies in Medicine, Tehran University of Medical Sciences, Tehran, Iran
3 Laboratory of Pharmaceutical Technology, Department of Pharmaceutics, Ghent University, Ottergemsesteenweg 460, 9000 Ghent, Belgium; joris.lammens@ugent.be (J.L.); Chris.Vervaet@ugent.be (C.V.)
4 Center for Advanced Light Microscopy, Ghent University, 9000 Ghent, Belgium
5 Laboratory of Pharmaceutical Process Analytical Technology (LPPAT), Department of Pharmaceutical Analysis, Ghent University, Ottergemsesteenweg 460, 9000 Ghent, Belgium; Thomas.DeBeer@Ugent.be
6 Department of Molecular Medicine, School of Advanced Technologies in Medicine, Tehran University of Medical Sciences, Tehran, Iran; e_motevaseli@tums.ac.ir
7 Department of Toxicology and Pharmacology, Faculty of Pharmacy, Tehran University of Medical Sciences, Tehran, Iran; mhghahremani@tums.ac.ir
8 Skin and Stem Cell Research Center, Tehran University of Medical Sciences, Tehran, Iran; mansorip@sina.tums.ac.ir
* Correspondence: refaridi@sina.tums.ac.ir (R.F.-M.); Kevin.Braeckmans@UGent.be (K.B.)

Table S1. Correlation between the effective siRNA concentration and the corresponding AuNPs concentration.

siRNA Concentration	AuNPs/mL [1]
5 nM	2.62×10^{10}
10 nM	5.25×10^{10}
15 nM	7.87×10^{10}
20 nM	1.05×10^{10}
30 nM	1.57×10^{11}
40 nM	2.10×10^{11}

[1] The concentration of AuNPs in the dispersions was estimated from their extinction spectra as previously reported (64).

Table S2. siRNA Sequences.

	Sense	Anti-sense
**Egln 1.13.1**	5′-CUAUUGUUCAUGUAAAACAUGAAAA-3′	5′-UUUUCAUGUUUUACAUGAACAAUAGCA-3′
**Egln 1.13.2**	5′-GCGUGACAUGUAUAUAUUAUCUAAA-3′	5′-UUUAGAUAAUAUAUACAUGUCACGCAU-3′
**Egln 1.13.3**	5′-CGCCAAGGUAAGUGGAGGUAUUCTT-3′	5′-AAGAAUACCUCCACUUACCUUGGCGUC-3′

Table S3. Primer Sequences used in Real-time PCR.

	Forward Primer	Reverse Primer	Size (bp)
**PHD-2**	5′-GCGTAACCCTCATGAAGTACA-3′	5′-CAACCCTCACACCTTTCTCAC-3′	128
**HIF-1α**	5′-GCTCACCATCAGTTATTTACGTG-3′	5′-CCGTCATCTGTTAGCACCAT-3′	138
**VEGF**	5′-CCGAAACCATGAACTTTCTGC-3′	5′-GACTTCTGCTCTCCTTCTGTC-3′	115
**FGF-2**	5′-GTCAAACTACAACTCCAAGCAG-3′	5′-GAAACACTCTTCTGTAACACACTT-3′	129
**β-Actin**	5′-GATTACTGCTCTGGCTCCTAG-3′	5′-GACTCATCGTACTCCTGCTTG-3′	147

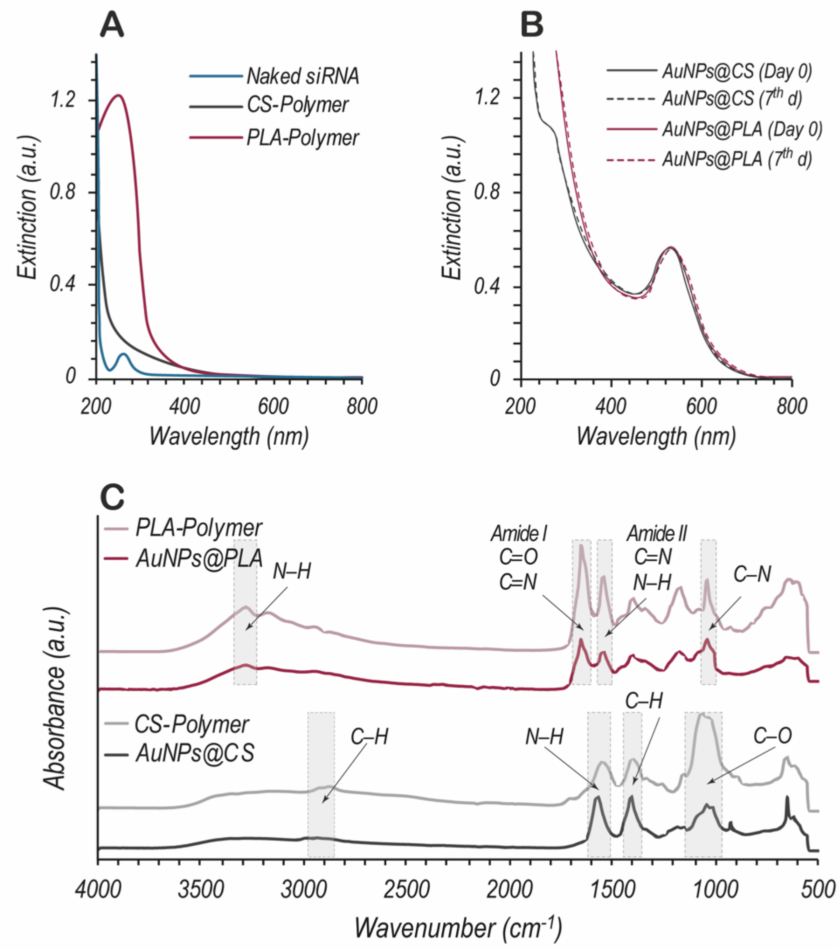

Figure S1. Characterization of layer-by-layer (LbL) synthesis with CS or PLA as outer layer. (A) UV-vis spectrum of Chitosan and Poly L-Arginine. (B) UV-vis spectrum of AuNPs@CS and AuNPs@PLA immediately after synthesis (day 0) and 7 days after synthesis. (C) FTIR spectra of Chitosan, AuNPs@CS, Poly L-arginine and AuNPs@PLA confirming successful capping of the NPs by CS or PLA as 3rd layer.

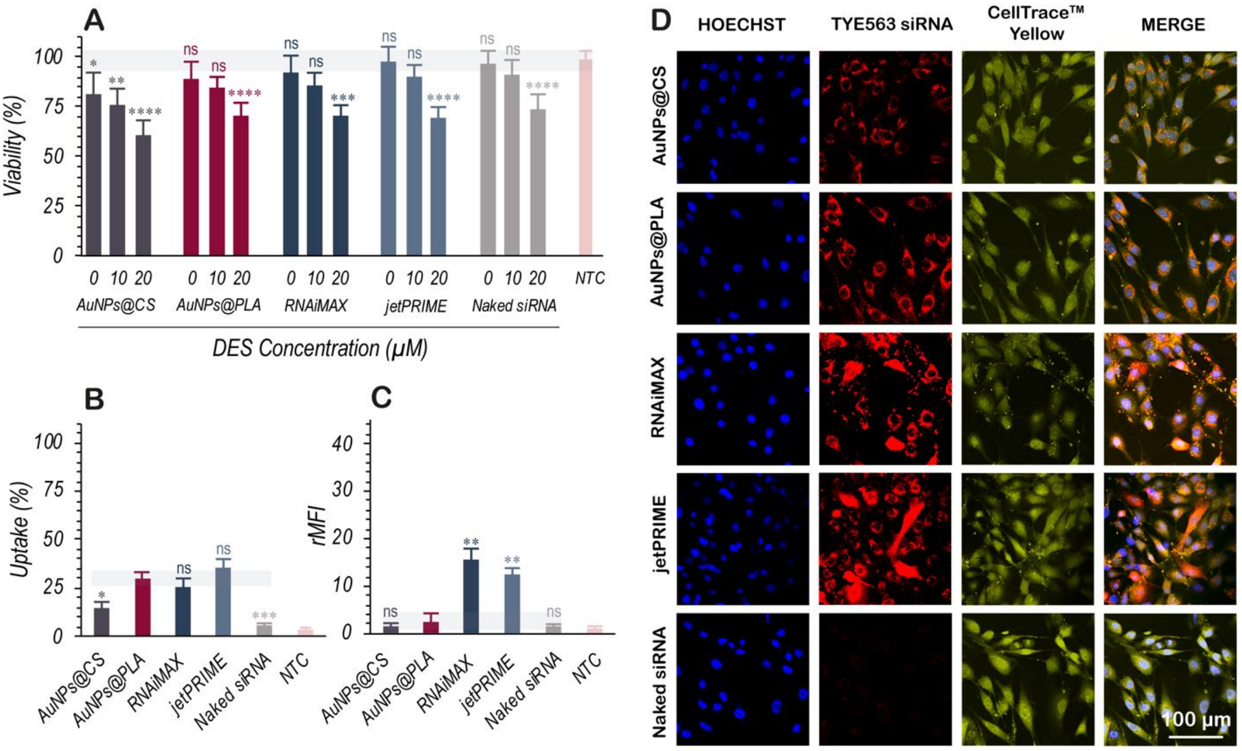

Figure S2. Cytotoxicity and evaluation of nanoformulation internalization in NIH-3T3 cells. (**A**) Cell viability of NIH-3T3 cells after 4 h incubation with the nanoformulations and transfection reagents (10 nM effective concentration of siRNA), washing and 20 h incubation with DES (0, 10, and 20 μM). (**B**) Uptake percentage (= % positive cells) and (**C**) relative mean fluorescence intensity per cell of TYE563 siRNA labeled nanocarriers (10 nM) after 4 h treatment on NIH-3T3 cells as determined by flow cytometry. (**D**) Confocal images of NIH-3T3 cells after 4 h treatment with different nanocarriers at 10 nM effective concentration of siRNA. Nuclei were stained with Hoechst 33342 (blue), the cytoplasm with CellTrace™ Yellow (yellow), while TYE563 siRNA is shown in red. The scale bar corresponds to 100 μm. Data are represented as the mean ± the standard deviation for a minimum of three independent experiments. Statistical significance is indicated when appropriate, in (A) when referring to not treated control (NTC) and in (B) and (C) when compared to AuNPs@PLA. (ns = not significant $p > 0.05$, * $p \leq 0.05$, ** $p \leq 0.01$, *** $p \leq 0.001$, **** $p \leq 0.0001$).

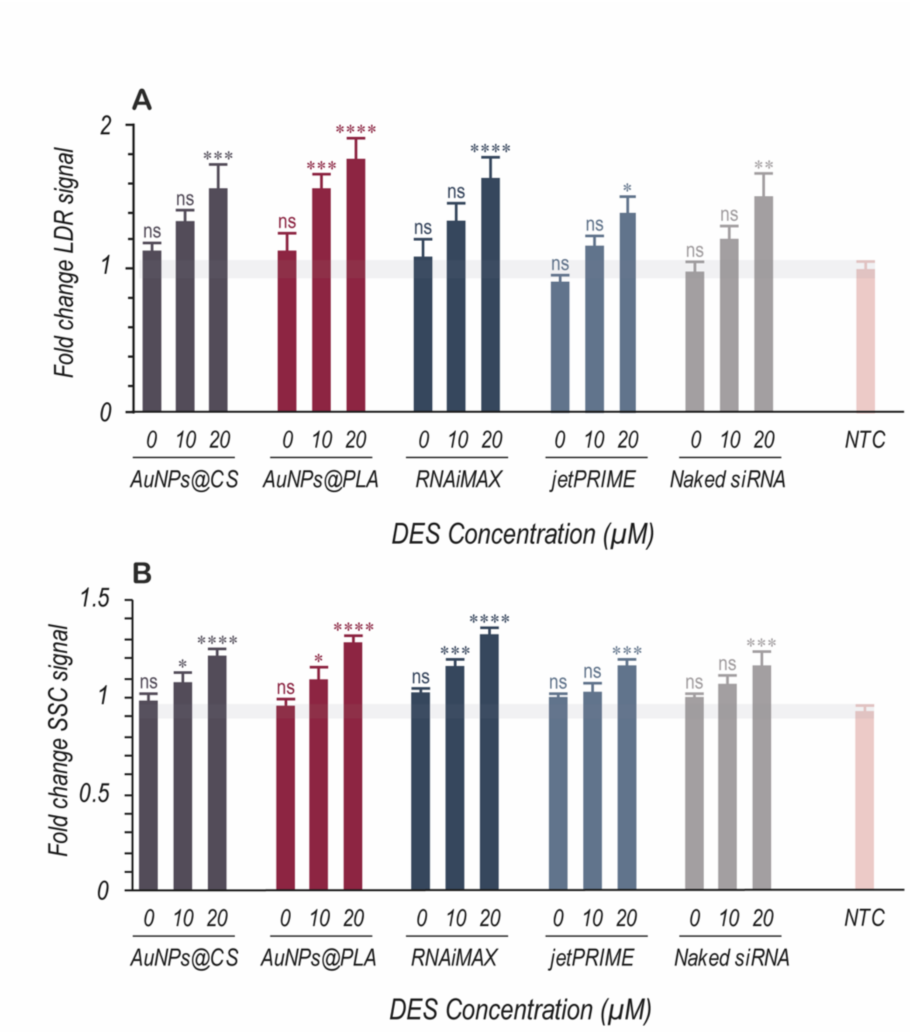

Figure S3. The effect of DES on lysosomal swelling and cellular granularity. (**A**) Fold change in LysoTracker® Deep Red (LDR) signal and (**B**) Fold change in side scatter (SSC) signal was measured via flow cytometry for NIH-3T3 cells treated 4 h with nanocarriers (10 nM effective concentration of siRNA) followed by 20 h treatment with 3 different concentrations of DES (0, 10, and 20 μM). Data are represented as the mean ± the standard deviation of three independent experiments. Statistical significance, with respect to the not treated control (NTC), is indicated when appropriate (ns = not significant $p > 0.05$, * $p \leq 0.05$, ** $p \leq 0.01$, *** $p \leq 0.001$, **** $p \leq 0.0001$).

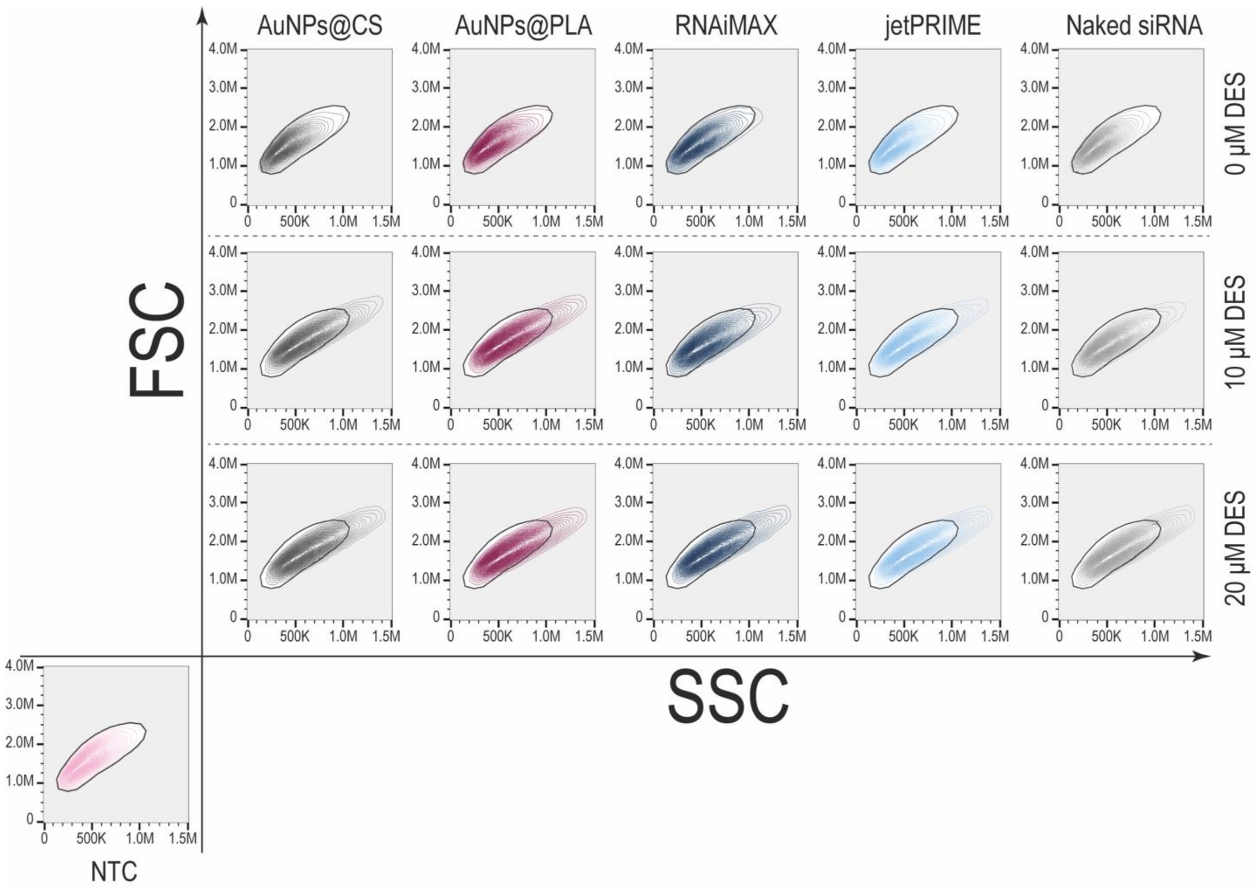

Figure S4. The effect of DES on cellular granularity. Representative contour plots for the not treated control (NTC) cells, or cells transfected with nanocarriers (30 nM effective concentration of siRNA) followed by 0, 10 and 20 µM DES treatment.

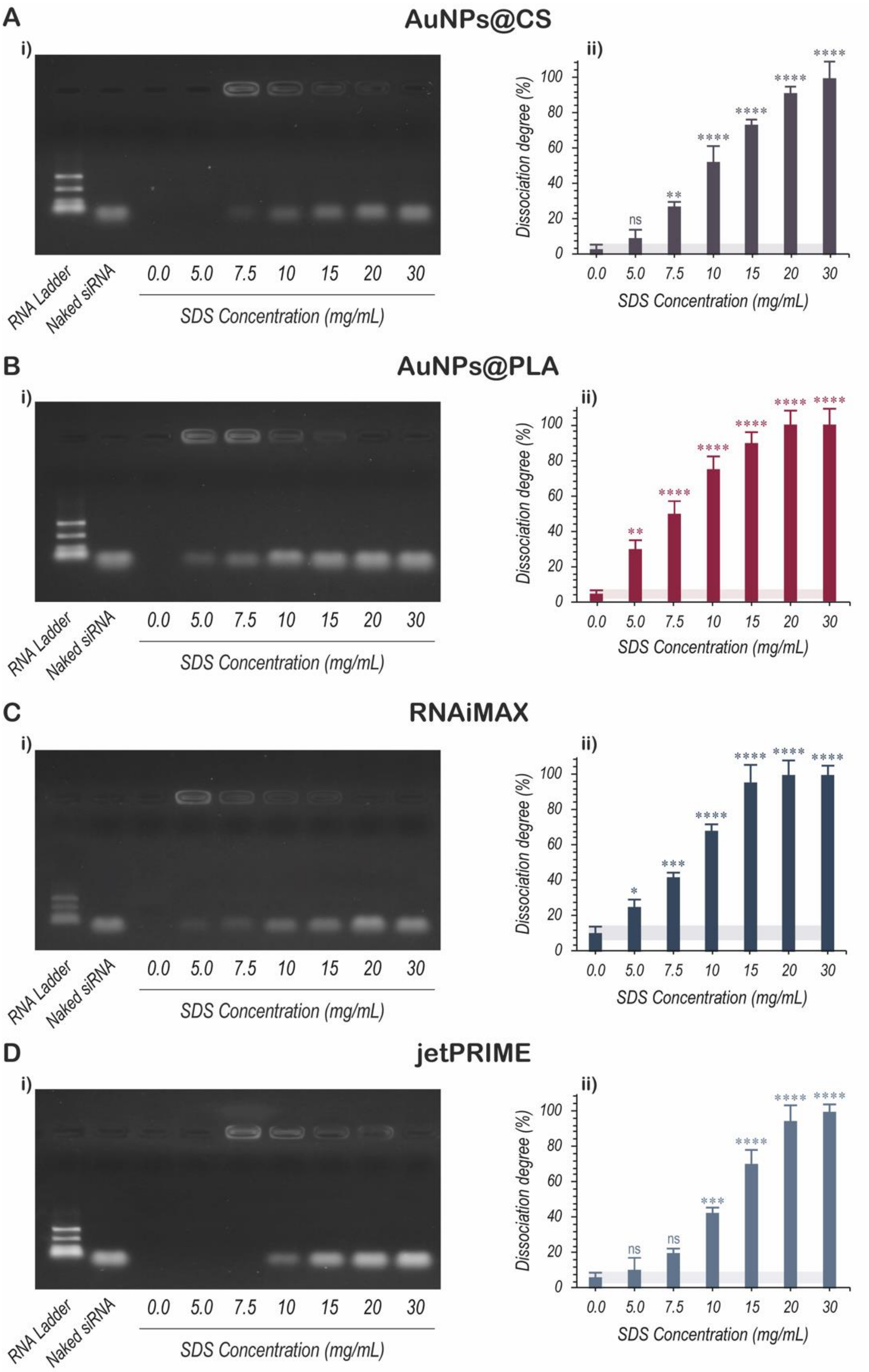

Figure S5. Forced dissociation of siRNA from nanoformulations in HEPES buffer by addition of SDS. Representative (i) gel electrophoresis images and (ii) the dissociation degree (%) calculated from FFS for (**A**) AuNP@CS, (**B**) AuNP@PLA, (**C**) Lipofectamine® RNAiMAX and (**D**) jetPRIME® with increasing concentrations of SDS.
